# 

**Published:** 1869-09

**Authors:** 


### PERSONAL.

Dr. W. W. Allport, who is so well and favorably known to the profession throughout this country, and in other countries too, where there are Dentists; recently sailed for Europe, to be absent for a few months. His object is physical recuperation and enjoyment. The Dr. by close confinement and hard work has made serious inroads upon his health. Our sincere desire is that the journey may be full of pleasure, and that he return as soon as practicable restored to health and vigor. Dr. A. is one of the men we can not afford to loose for any considerable time.

### The Dental Register

### OF THE WEST.

*Issued Monthly, at $3 a Year in Advance.*

DEVOTED TO THE INTERESTS OF THE DENTAL PROFESSION.

EDITED BY J. TAFT AND G. WATT.

The volume commences in January and closes in December.

The Register being issued monthly is always fresh, therefore indispensable to the practitioner who desires to keep posted up with the new inventions and improvements which are daily developed. Neither expense nor effort will be spared to give value and variety to its contents. Articles will be illustrated with well executed engravings whenever they will add to the interest or assist in explanation.

Its extensive circulation and frequency of issue makes it one of the BEST DENTAL ADVERTISING MEDIUMS in the United States.

RATES OF ADVERTISING.

One page, 1 year, $50. Half page, 1 year, $30. Quarter page, or card, 1 year $20.

All advertising bills payable in advance, or at furthest after the issue of the first number containing the advertisement. All transient advertisements to be paid for in advance.

☞ CONTRIBUTIONS SOLICITED.

Address, J. TAFT, or "DENTAL REGISTER," Cincinnati Ohio.

Business Communications to

**J. TAFT, Publisher,**

No. 117 West Fourth St.

